# Constraints upon the Response of Fish and Crayfish to Environmental Flow Releases in a Regulated Headwater Stream Network

**DOI:** 10.1371/journal.pone.0091925

**Published:** 2014-03-19

**Authors:** Edwin T. Chester, Ty G. Matthews, Travis J. Howson, Kerrylyn Johnston, Jonathon K. Mackie, Scott R. Strachan, Belinda J. Robson

**Affiliations:** 1 Environmental and Conservation Sciences, Murdoch University, Perth, Western Australia, Australia; 2 School of Life and Environmental Sciences, Deakin University, Warrnambool, Victoria, Australia; 3 Marine and Freshwater Research Laboratory, Murdoch University, Perth, Western Australia, Australia; The Australian National University, Australia

## Abstract

In dry climate zones, headwater streams are often regulated for water extraction causing intermittency in perennial streams and prolonged drying in intermittent streams. Regulation thereby reduces aquatic habitat downstream of weirs that also form barriers to migration by stream fauna. Environmental flow releases may restore streamflow in rivers, but are rarely applied to headwaters. We sampled fish and crayfish in four regulated headwater streams before and after the release of summer-autumn environmental flows, and in four nearby unregulated streams, to determine whether their abundances increased in response to flow releases. Historical data of fish and crayfish occurrence spanning a 30 year period was compared with contemporary data (electrofishing surveys, Victoria Range, Australia; summer 2008 to summer 2010) to assess the longer–term effects of regulation and drought. Although fish were recorded in regulated streams before 1996, they were not recorded in the present study upstream or downstream of weirs despite recent flow releases. Crayfish (*Geocharax* sp. nov. 1) remained in the regulated streams throughout the study, but did not become more abundant in response to flow releases. In contrast, native fish (*Gadopsis marmoratus*, *Galaxias oliros, Galaxias maculatus*) and crayfish remained present in unregulated streams, despite prolonged drought conditions during 2006–2010, and the assemblages of each of these streams remained essentially unchanged over the 30 year period. Flow release volumes may have been too small or have operated for an insufficient time to allow fish to recolonise regulated streams. Barriers to dispersal may also be preventing recolonisation. Indefinite continuation of annual flow releases, that prevent the unnatural cessation of flow caused by weirs, may eventually facilitate upstream movement of fish and crayfish in regulated channels; but other human–made dispersal barriers downstream need to be identified and ameliorated, to allow native fish to fulfil their life cycles in these headwater streams.

## Introduction

Much has been published on the consequences of river regulation for populations of fish and other large consumers, however much less work has focussed on the impacts of regulating headwater streams [1]. In semi–arid and mediterranean climate zones, first to third order streams are often regulated for water extraction [2], and this may lead to perennial streams becoming intermittent or the prolongation of dry periods in intermittent streams [3], [4]. Regulation of headwater streams for water extraction often involves the construction of small weirs, which act as barriers to the movement of freshwater animals [4]. Therefore, such regulation may have a negative impact on abundances by two mechanisms: reducing habitat area through streambed drying downstream of the weir, and by creating barriers to movement.

Biodiversity in seasonally flowing streams largely depends on the viability and persistence of refuges (such as pools) within the channel, and upon a cycle of recolonization from perennially flowing parts of the stream or the greater catchment [5]. Maintaining drought refuges is one key action that can sustain biodiversity in seasonally flowing streams [6]. Flow releases from weirs could be targeted to sustain refuge pools, by increasing the total duration of flow downstream during the year [4], and through selective shorter–term releases. Connectivity through water flow down the stream channel allows colonization and movement downstream from perennial reaches (especially from above weirs) and upstream from within the greater catchment. Fish species found in headwater streams can recolonise streams quickly following disturbances like drought [7]. Their (generally) small bodies, short life spans, and early–aged reproduction (e.g. Galaxiidae species mature in their first year of life and have the highest gonadosomatic index of all Australian fish) permit rapid population growth [8], facilitating that process, but even small barriers may inhibit dispersal [9]. The benthic native crayfish may disperse more slowly than fish, but channel obstructions are less likely to be barriers because they may travel overland for short distances.

Flow releases are a commonly used tool for managing stream and river health; however there are relatively few evaluations of their success in non–perennial streams (but see [10], [11]). Such evaluations are required for adaptive management, to improve the targeting of flows for maximum benefit. Here, we studied summer flow releases from weirs in headwater streams and their effect on fish and crayfish assemblages. There were three reasons why we expected a positive response to summer flow releases: (1) increased flows may increase the connectedness of streams and facilitate movement; (2) summer releases may sustain refuge pools by improving water depth, water quality and survivorship; (3) summer releases may keep riffles located downstream of weirs wetted for longer, providing a greater duration of spawning habitat for some species (e.g. *G. oliros* [8]). Therefore, the aim of this project was to determine whether native fish and crayfish responded to these releases in headwater streams of the Victoria Range, Australia, where stream regulation for potable use is common practise. To do this, species assemblages were sampled in four regulated streams and four unregulated streams, before and after the initiation of summer flow releases.

## Methods

### Ethics statement

All research in this study was conducted under permits from the Department of Sustainability and Environment and the Department of Fisheries, Victoria (DSE permit number 10003269, DPI permit number RP896). Electrofishing was carried out in accordance with the Australian Code of Electrofishing Practice (1997) and Australian Code of Practice for the Care and Use of Animals for Scientific Purposes (7th edition 2004) under approval by Deakin University Animal Welfare Committee (A15-2007). All streams and species sampled are protected within the Grampians National Park, Victoria, Australia. All captured animals were returned to the streams alive and no mortality was recorded. Data may be obtained by contacting the corresponding author.

### Study sites and flow regimes

**Figure 1:**
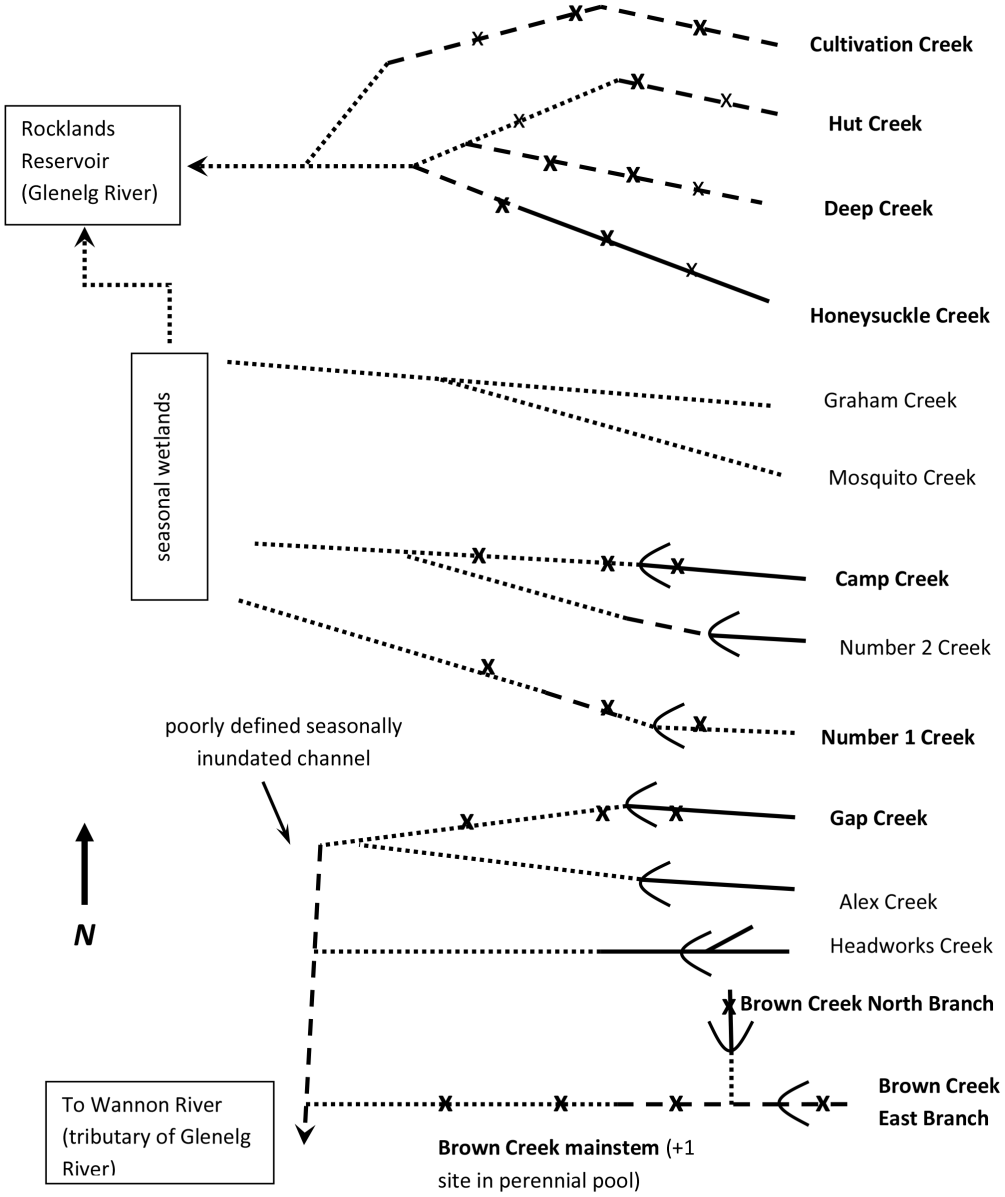
Diagram of the study streams (named in bold) in the Victoria Range, showing flow regimes and representing conditions at peak dryness, without summer passing flows (solid lines  =  perennial flow, dashed lines  =  seasonal flow with perennial pools and dotted lines  =  seasonal, completely dry) and weirs (‘U’ shapes). Survey sites denoted by ‘**x**’: primary sites were upstream and downstream of weirs, and at similar elevations on unregulated streams; all potential fish/crayfish refuges were sampled. Sites with faint ‘x’ were in surveys prior to this study, and were visited in summer 2008; they were subsequently dropped as they were not needed to characterize the unregulated streams for comparison with the regulated ones. General flow direction is from right to left. Note that this is a schematic diagram and is not to scale.

The Victoria Range (maximum altitude 979 m) is the westernmost of a series of mountain ranges running north–south in the Grampians National Park in western Victoria, Australia. With a mediterranean to semi–arid climate (400–600 mm average annual rainfall), streams vary from perennially flowing to seasonally dry with considerable interannual variation in flow regime [5]. Riparian vegetation is dry sclerophyll woodland and forest, and all streams have sandstone boulders and cobbles with areas of bedrock, descending to a sandplain where channels may become indistinct (described in [6]) (Figure 1). From 2000 to 2010, stream flows were affected by one of the longest and most intense droughts historically experienced [12]. Compared to historical norms, flow periods were generally shorter, with a prolonged dry summer–autumn, and reduced spring rains.

Four of the eight study streams are regulated for town water supply (Figure 1), with weirs that extract water and deliver it to storage via underground pipes (described in [4]). Four of the streams have perennial flow: Honeysuckle Creek (unregulated), Gap Creek (regulated in 1914 [13]) and Camp Creek (regulated in 1960 [13]), which both have intermittent flow downstream of their weirs, and Brown Creek (regulated in 1939 [13]), which has two branches that are regulated. The other four streams flow seasonally: Cultivation Creek, Deep Creek and Hut Creek (unregulated with perennial pools over summer–autumn) and Number 1 Creek (regulated and can dry completely in summer–autumn). Further descriptions of the streams, stream locations and flow regimes may be found in [3] and [5].

An environmental flow release of up to 0.4 ML/day was released into Camp and Gap Creeks during April 2009 and in both branches of Brown Creek: increased flows were passed through the weirs after modifications for that purpose. These comprised valves in ∼8 cm diameter pipes, installed above the base flow height downstream of the weirs, and not specifically designed to allow animal movement. The passing flows were continued until May 2009 in all creeks except for Camp Creek, which continued until early June; however, there was no interruption in flow in Camp Creek due to the contemporaneous onset of winter rain. In contrast, cessation of the passing flows in May led to a short period of interrupted flow (and dry stream beds) directly below the weirs in Gap Creek and both branches of Brown Creek. Gap Creek did not resume flow until early June 2009, from diffuse sources after winter rain; Number 1 Creek also started flowing at this time (it did not receive an environmental flow release during April 2009, because there was no water upstream of the weir). Temporally patchy flow also resumed in parts of Brown Creek during June, but again not in reaches immediately downstream of the weirs, which only held water following spates later in winter. Passing flow releases began again at the end of September 2009 in all four regulated streams and continued into winter 2010 (but again, there was no water upstream in Number 1 creek from early summer).

### Study species

Historically, river blackfish (*Gadopsis marmoratus* (Richardson)), obscure galaxias (*Galaxias oliros* Raadik) [14], and common jollytails (*Galaxias maculatus* (Jenyns)) were the native fish species recorded most often in Victoria Range streams [15]. *G. marmoratus* in these streams is probably the as yet undescribed northern species [16]. The southern pygmy perch (*Nannoperca australis* Günther) and the eastern dwarf galaxias (*Galaxiella pusilla* (Mack)) have been recorded in vegetated downstream reaches and in riverine wetlands [15]. These *Galaxiella* are probably the western, undescribed species [17]. Exotic fish are rare in these streams, but the redfin perch (*Perca fluviatilis* Linnaeus) has been recorded in low numbers in Cultivation Creek [15]. An undescribed species of freshwater crayfish (*Geocharax* sp. nov.1) also occurs commonly in these streams [5], [18]. This assemblage is typical of western Victorian upland streams and is less rich in fish species (including of exotic species) than lowland streams [19] and rivers [20].

### Sampling design

The hypotheses tested in this study were: (1) native fish are less abundant in regulated streams than unregulated streams; (2) fish occur less frequently in regulated streams receiving summer passing flows than in nearby unregulated streams; (3) species composition (including crayfish) differs between regulated streams receiving passing flows during summer and nearby unregulated streams. Fish and crayfish were sampled over two years (from summer 2008 to summer 2010) to compare four regulated streams (Gap Creek, No. 1 Creek, Camp Creek, Brown Creek) with four unregulated streams (Cultivation Creek, Hut Creek, Deep Creek, Honeysuckle Creek) using single pass electrofishing surveys.

Electrofishing surveys were done in summer (March) and spring (October) 2008 to provide “before treatment” data; then in spring (October) 2009, and summer (March) 2010, after passing flows had been in operation (Table 1). These sampling times should have detected both fish migrating in spring (most likely spawning galaxias) and fish and crayfish that had remained in refuge pools further downstream over the summer. Individuals were counted and, in the summer sampling, measured (total length and weight (g) for fish, occipital carapace length (OCL) for crayfish); crayfish were not always netted, so estimates include counts for animals seen and not captured.

**Table 1:** Presence of fish and crayfish in the eight streams at each sampling time, compared with historical presences.

Stream	Type	Historical (1979, 1989, 1995-96) (2006-2007)^#^	Summer 2008	Spring 2008	Spring 2009	Summer 2010
**Brown Ck**	**Regulated**	*Geocharax, G. marmoratus, G. oliros*	*Geocharax*	*Geocharax*	*Geocharax*	*Geocharax*
**Gap Creek**	**Regulated**	*Geocharax, G. oliros*	*Geocharax*	*Geocharax*	*Geocharax*	*Geocharax*
**No. 1 Creek**	**Regulated**	*Geocharax, G. oliros*	*Geocharax*			
**Camp Creek**	**Regulated**	*Geocharax, G. marmoratus, G. oliros*	*Geocharax*			*Geocharax*
**Hut Creek**	**Unregulated**	*Geocharax#, G. marmoratus, G. oliros, G. maculatus*	*Geocharax, G. oliros, G. maculatus, N. australis*	*Geocharax*	*Geocharax*	*Geocharax, G. oliros, G. maculatus*
**Cultivation Ck**	**Unregulated**	*Geocharax#, G. marmoratus, G. oliros, G. maculatus, P. fluviatilis*	*Geocharax, G. marmoratus, G. oliros, G. maculatus, N. australis, G. pusilla*	*Geocharax, G. marmoratus, G. oliros, G. maculatus*	*Geocharax, G. marmoratus, G. oliros, G. maculatus*	*Geocharax, G. marmoratus, G. oliros, G. maculatus, P. fluviatilis*
**Deep Creek**	**Unregulated**	*G. marmoratus#, G. oliros*	*Geocharax, G. marmoratus, G. oliros, G. maculatus, N. australis*	*G. marmoratus*	*G. marmoratus*	*G. marmoratus, G. oliros, G. maculatus*
**Honeysuckle Ck**	**Unregulated**	*Geocharax#, G. marmoratus#, G. oliros, G. maculatus, N. australis*	*Geocharax, G. marmoratus, G. oliros, G. maculatus, N. australis*	*G. marmoratus, G. oliros*	*G. marmoratus, G. oliros*	*G. marmoratus, G. oliros*

Compiled from Raadik (1996) and Jackson and Davies (1983) as well as author's unpublished data from 2006–2007. Bold indicates species found upstream of weirs; underlined indicates rare or encountered infrequently (i.e. very patchily distributed, in one place in channel: not given for historical occurrences); ^#^ denotes almost all animals caught electrofishing the unregulated streams in 2007 were these species.

Electrofishing equipment was customized, based around a SAMUS 725M 12 volt unit fitted into a ruggedized, wearable vest with additional safety features and output meters for the operator. An 11” (280 mm) circular stainless steel anode was used, along with a 3 m cathode of 6 mm woven stainless steel cable. Waveform was varied systematically through the survey, to target animals (and species) of different size and sensitivity, while minimizing error from alarmed individuals escaping detection as well as the chance of mortality. Settings (frequency 99 Hz, pulse width 0.05–0.25 ms) were based on previous trials with this equipment, and took into account the very different reactions of fish and crayfish. All sites had relatively narrow (1–5 m) and shallow channels with substantial woody debris, and riffle/run–pool sequences which could be efficiently searched by the team of three operators as they moved upstream. However, since streams varied in width, depth, presence of pools and woody debris, and intermittency, total effort in each stream also varied to maintain a consistent sampling efficiency. At all reaches, care was taken to maximize the probability of detection of any animals present, whether in a flowing run or in isolated refuge pools.

Hundred metre reaches — a substantial part of these short streams, and in some cases most that was accessible — were sampled upstream and downstream of weirs in regulated streams, and at similar positions in the catchment in unregulated streams (Figure 1). Additionally, and because no fish were initially found in the regulated streams, reaches were also sampled well downstream of the weirs to exclude the possibility that they were present but had not made their way upstream. There are weirs on two branches of Brown Creek, so both were sampled, as well as a near perennial spring–fed pool between the confluence and the downstream site. Two sites were sampled in each unregulated stream after 2009, apart from the smaller Hut Creek where only one reach, in the most reliably flowing section, was sampled (so, total 25 sites in 2008, 20 in 2009, Figure 1). Confidence in the ability to characterize these streams was bolstered by the consistency of results compared with the 2007 pilot study, which had shown a high degree of distinctiveness among them. For these reasons, total linear metres sampled (as measured by GPS, and surveyed along the bank) differed somewhat among streams; therefore, total catch was standardized to 100 m for comparison.

Historical data on fish and crayfish distributions (presence/absences) from [13], [15] and also Tunbridge (1989; as reported in 13) were compared with our data to determine longer term temporal changes in populations (Table 1). Jackson and Davies [15] used a range of gear including electrofishing (number of passes not specified), dip–netting in backwaters and gill nets in deep pools and surveyed sites between 30 and 60 m in length in spring and early summer, 1979. Raadik [13] used single pass electrofishing and occasional dip–netting in 1995 and 1996 and sampled stream lengths up to 100 m. We had collected data on fish and crayfish presences prior to 2008 using baited traps, while sampling invertebrates, and from initial (winter 2007) electrofishing in unregulated streams. While there are some methodological variations among these studies, they provide definite presence records of fish and crayfish species in Victoria Range streams over three decades. This provided a context in which to assess the effects of environmental flow releases.

### Data analysis

Hypothesis 1 and 2 could not be formally tested because no fish were captured in the four regulated streams. That is, obtaining zeroes for half of the cells in the design meant that formal statistics could not be used. This result does provide clear confirmation of both hypotheses because fish were collected in large numbers in all four unregulated streams (see below), demonstrating the efficacy of the fish capture method and therefore the veracity of the zero values. Hypothesis 3, that species composition would differ between the regulated and unregulated streams was therefore also confirmed without further need for statistical testing, although the crayfish *Geocharax* sp. nov. 1 was present in the regulated streams.

Differences in the fish/crayfish assemblage composition among unregulated streams were analysed using fish counts per 100 m from each stream, untransformed, in a two factor ANOSIM without replication: stream (n = 4) by time (n = 4: summer 2008, spring 2008, spring 2009, summer 2010) in PRIMER version 6 [21]. Chi-square tests were used to determine whether crayfish size frequencies (OCL, n = 5: <10 mm, 11–15 mm, 16–20 mm, 21–25 mm, >26 mm) differed among streams (counts per 100 m, n = 5, Hut Creek, Brown Creek east, Brown Creek north, Brown Creek mainstem, Gap Creek). Chi-square tests for homogenous counts of crayfish were used to determine whether crayfish responded to flow releases in Gap Creek and Brown Creek, separately, by comparing capture frequency per 100 m across the four sampling times.

## Results

### Species occurrences: flow releases and historical data

There are four clear results from this dataset. Firstly, although fish were recorded in the regulated streams prior to 1996, no fish had been recorded upstream of weirs in any streams and only a single fish (*G. marmoratus)* was observed downstream of the weir in Camp Creek in winter 2006 (Table 1). Secondly, fish were consistently present in unregulated streams (except Hut Creek in spring 2008 and summer 2009) despite prolonged drought conditions during 2006–2010. Thirdly, flow releases in 2010 were not associated with the presence of fish species in regulated streams, because no fish were detected in any of the regulated streams (Table 1). Fourthly, the crayfish *Geocharax* sp. nov. 1 remained in the regulated streams throughout the study, and occupied reaches downstream of weirs that were inundated by the flow releases. However, its occurrence in streams where it was already rarely detected before summer flow releases had not changed by 2010.

Several species were rare and only occasionally encountered in the unregulated streams (Table 1): the pygmy perch (*Nannoperca australis*), the dwarf galaxias (*Galaxiella pusilla*) and the non-native redfin perch (*Perca fluviatilis)*. The latter was also captured in the same stream (Cultivation Creek) in 1979 [15]. These occurrences may be partly explained by the greater effort (i.e. more sites) in summer 2008, although the *P. fluviatilis* was caught in 2010.

### Patterns of abundance in fish and crayfish

**Figure 2:**
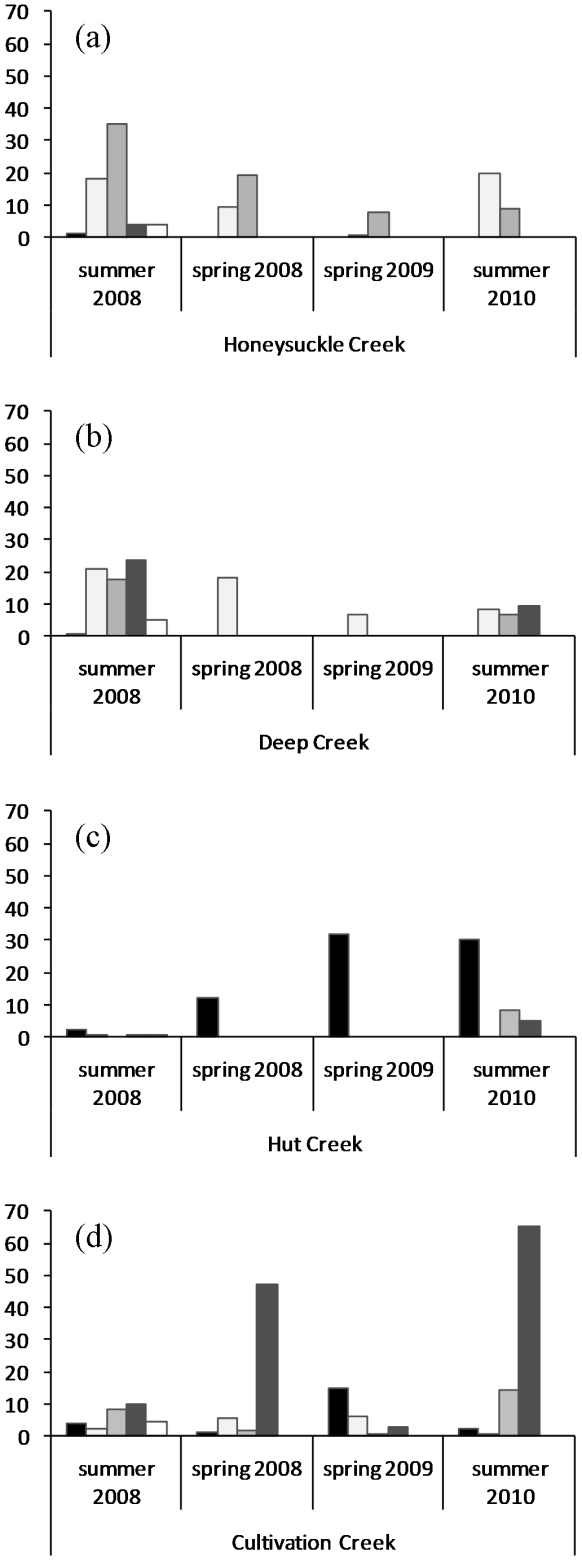
Total abundances per 100(a) Honeysuckle Creek; (b) Deep Creek; (c) Hut Creek; (d) Cultivation Creek. Black  =  *Geocharax* sp., pale grey  =  *G. marmoratus*, mid-grey  =  *G. oliros*, dark grey  =  *G. maculatus*, white  =  *N. australis*.

The four unregulated streams differed markedly in species composition and abundance (R = 0.5, *P* = 0.008; Figure 2) but assemblage composition did not differ consistently among sampling times, because the stream to stream variation led to there being greater levels of variation within times than between them (R = −0.18, *P* = 0.87). *G. marmoratus* were most abundant in Honeysuckle and Deep Creeks but also occurred in Cultivation Creek and Hut Creek; those in Cultivation Creek were restricted to a single refuge pool sampled upstream. *G. oliros* was most abundant in Honeysuckle Creek but also occurred in the other three streams. *G. maculatus* was rare in all creeks except Cultivation Creek where it occurred in large numbers (Figure 2d). While the two *Galaxias* species were more abundant in summer 2008 than summer 2010 in Deep and Honeysuckle Creeks (i.e. those with the most perennial water), Cultivation and Hut Creeks (which dried to only a few pools over this period) held more in 2010, although there were many *G. maculatus* in Cultivation Creek in spring 2008 when it was flowing. Very few juvenile *G. maculatus* were recorded (Table 2). Adult *G. oliros* were widespread in the unregulated streams but again, juveniles were rare (Table 2). However, juvenile *G. marmoratus* were relatively common in Honeysuckle and Deep Creeks (Table 2).

**Table 2:** Numbers of adult and juvenile fish and crayfish recorded for each stream in summer, per 100

			*Geocharax* sp. nov. 1		*G. marmoratus*		*G. oliros*		*G. maculatus*		*N. australis*	
Regulation	Streams	Sampling period	Adults > 15 mm	Juveniles < 15 mm	Adults > 150 mm	Juveniles < 123 mm	Adults > 42 mm	Juveniles < 42 mm	Adults > 38 mm	Juveniles < 38 mm	Adults > 30 mm	Juveniles < 30 mm
**Unregulated**	Hut Creek	summer 2008	0.4	2.0	0	0.8	0	0	0.4	0	0.4	0
		summer 2010	26.0	4.0	0	0	8	0	5.0	0	0	0.0
	Cultivation Creek	summer 2008	2.7	1.3	1.7	0.7	7.3	1	9.7	0	1.0	3.7
		summer 2010	2.0	0	0.5	0	14	0.5	64.5	0.5	0	0
	Deep Creek	summer 2008	0.3	0	15.7	5	17.7	0	23.3	0	5.0	0
		summer 2010	0	0	5.3	2.7	6.7	0	9.3	0	0	0
	Honeysuckle Creek	summer 2008	P	P	5.7	12.7	34	1	4.0	0	3.7	0
		summer 2010	0	0	3	17	8.5	0.5	0	0	0	0
**Regulated**	Brown Creek	summer 2008	5.6	5.6								
		summer 2010	7.8	7.8								
	Gap Creek	summer 2008	P	P								
		summer 2010	13.5	13.5								
	Number 1 Creek	summer 2008	P	P								
		summer 2010	0	0								
	Camp Creek	summer 2008	P	P								
		summer 2010	1.3	0.3								

Adult and juvenile fish sizes (*G. oliros* based on *G. olidus*) from [20], [30], [51]. Adult and juvenile crayfish sizes (OCL) for *Geocharax falcata* were used from [52]. P =  present, not measured (too few or seen and not caught).

**Figure 3:**
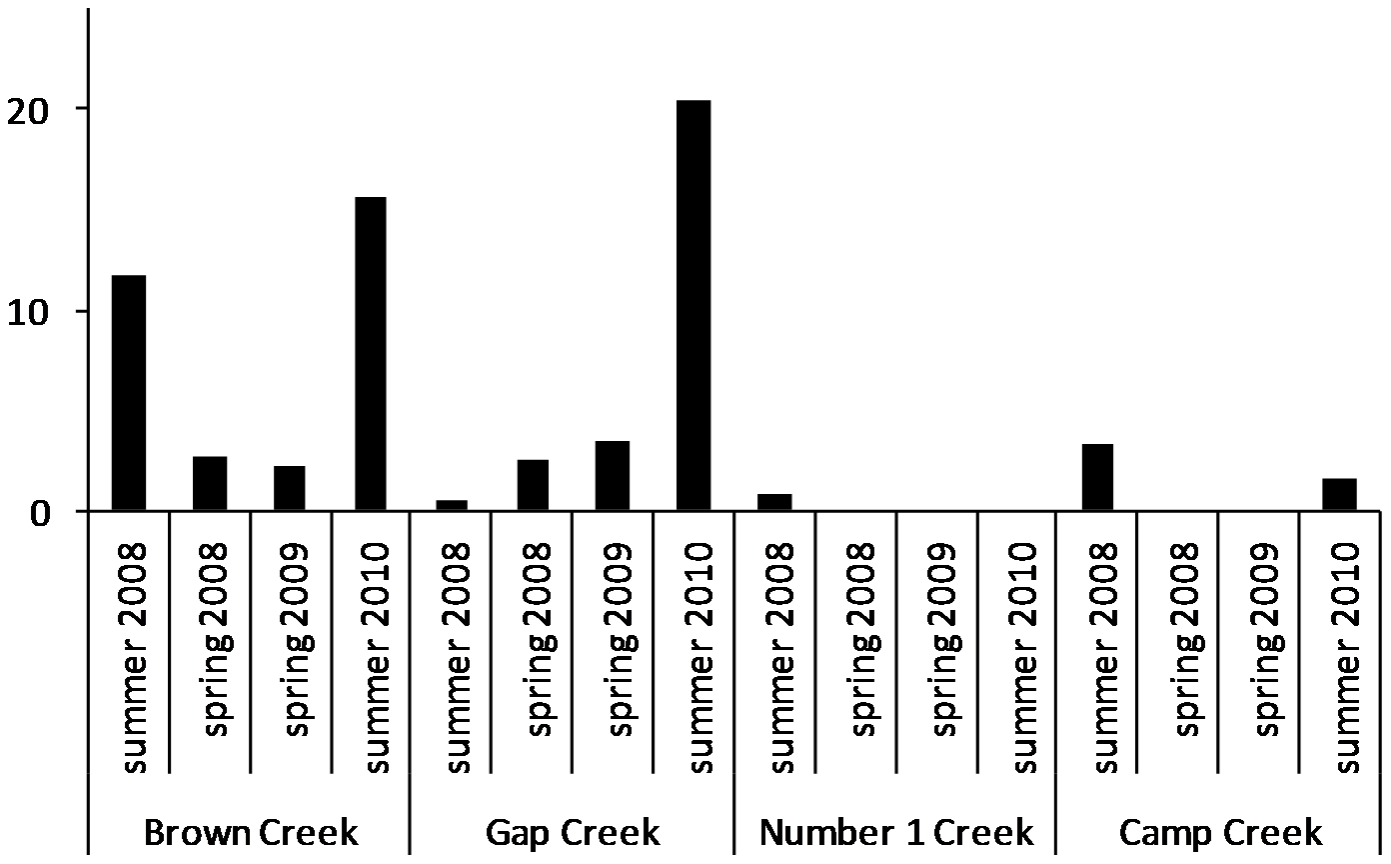
*Geocharax* sp. nov. 1 abundance for each stream per 100 m sampling effort.

A single crayfish *Geocharax* sp. nov. 1 was recorded from Deep Creek. Only a few were found in Honeysuckle Creek, in a pool well downstream (Figure 2a), and none were ever found upstream, although they had been recorded there in 1989 (Table 1). *Geocharax* occurred alone and in low abundances in regulated Number 1 and Camp Creeks (Figure 3). Pools upstream of weirs in Brown and Gap Creeks were refuges for crayfish, although they were not found upstream in Brown Creek in spring 2009.

Fish and crayfish co-occurred in unregulated streams, but rarely in significant numbers other than in Cultivation Creek. Crayfish were relatively abundant in Hut Creek, where fish were uncommon, and in spring 2008 and 2009 only crayfish were recorded there (Figure 2c). In contrast, crayfish were uncommon in Honeysuckle and Deep Creeks where fish abundances were highest, especially *G. marmoratus*. Where large crayfish populations were found, size frequency differed among streams (χ^2^_16_  = 47, *P*<0.01). Juveniles were rare in Hut Creek but frequent in Gap Creek, and Brown Creek (Table 2). *Geocharax* OCL ranged between 7–33 mm, and an increased proportion of crayfish of all sizes was captured following flow releases in Gap (χ^2^_3_  = 32.8, *P*<0.01) and Brown Creeks (χ^2^_3_  = 16.75, *P*<0.05; Figure 3).

## Discussion

### Responses to environmental flow releases by native fish and crayfish

These results clearly showed that a single year of environmental flow releases in headwater streams in the Victoria Range was insufficient to produce a response in fishes. Crayfish were detected in formerly dry streambeds downstream of weirs in Gap Creek and Brown Creek, but they had apparently moved only limited distances within each stream to occupy wetted channel close to source areas. The apparent disappearance of crayfish upstream of the weir in Brown Creek in spring 2009 was anomalous, and may have been due to animals dispersing into expanded habitat, or even because of increased predation from birds in a weir pool made shallow by flow release.

Macroinvertebrates responded quickly to these flow releases [4], but it is probable that a single year is too short a time or that the flows were of insufficient magnitude to allow native fish to recolonise the regulated streams. It is also possible that dispersal barriers downstream are also preventing recolonisation by fish. Such barriers may or may not be important for crayfish dispersal; but movement from refuges downstream may be slow, and terrestrial ‘shortcuts’ between streams would seem to be the quickest route to colonize streams like Number 1 and Camp Creeks, if survival on land is possible for *Geocharax*. However, there was no evidence of this happening up to 2010.

*Galaxias maculatus* is known to be a weak swimmer [9] but *G. oliros* appears to be more competent [22]. We have observed *G. oliros* in lower reaches of the Brown Creek system, but they were not observed downstream of the weirs during the present study. This suggests that some road crossings, fords and culverts, on the regulated streams may be impassable to small fish, even during flow releases. Recent research shows that it is possible to modify most culvert and road crossing designs to facilitate migration by small species of native fish [9], [23] and such modifications should be considered as an adjunct to environmental flow releases to improve ecosystem connectivity and ecological outcomes [24].

The weirs themselves are certainly substantial barriers, so fish absences upstream are not surprising. The only opportunity for fish to access channels upstream of weirs would have been during large rainfall events, across the weir wall. This is probably beyond the capabilities of the small–bodied native fish species found in headwater streams [25]. Also, flow releases approximately equivalent to baseflow (<0.4 ML/day/channel) at the weirs did not necessarily result in surface flows lower in the catchment, so there were probably still reaches of dry streambed at times that would limit upstream movement by fish [22]. Lastly, weirs tend to create three discrete levels of flow downstream (baseflow arising from the flow release, no flow, or weir–topping spates) in contrast to a more gradual transition between flow states in unregulated streams.

### Native fish populations in regulated streams declined, whereas crayfish remained

Clear patterns were evident in native fish occurrences and fish were either absent from the regulated streams or were present in such low numbers that they were not detected. Fish were neither observed nor collected upstream of weirs from 2006 to 2009, yet with the same sampling methods, large numbers of fish were collected in the unregulated streams from 2007 onwards, suggesting that if fish were present in the regulated streams, they would have been caught. Native fish appear likely to have become locally extinct in the regulated streams, not only upstream, but also downstream of weirs, in the decade between 1996 and 2006 and fish abundances had probably declined steadily in these streams prior to that time. Therefore, the hypothesis that native fish were less abundant in regulated streams than unregulated streams in the Victoria Range was confirmed. In contrast, populations of the crayfish *Geocharax* sp. nov. 1 did not decline in the regulated streams.

Hut Creek is unregulated but fish were not observed there between spring 2008 and spring 2009, showing that these headwater streams may be temporarily naturally fishless. *Galaxias oliros* were observed in Hut Creek both before (winter 2007) and after (summer 2010) this period, showing that fish populations in some unregulated streams were affected by the “millennium drought” [12], which peaked in 2006 in the Grampians [5], followed by recovery. Indeed, assemblages in the other three unregulated streams were consistent across sampling times and also consistent with historical records back to 1979.

The reasons for these patterns probably lie in the autecology of each species of fish and crayfish. Some crayfish species are able to walk overland in search of new suitable habitat when water bodies dry up [26], [27]. If *Geocharax* sp. nov. 1 has this ability, then constructed weirs may not present a barrier to their persistence in regulated streams (particularly when dispersing *downstream* from refuges in weir pools). Furthermore, *Geocharax* sp. nov. 1 is able to aestivate during dry periods in a chamber that it constructs in the stream bed [5] and so it is better able to resist drying than native fish. *Geocharax* reproduction does not involve migration and juveniles co-occur spatially with adults so life cycles can, as far as we know, be completed within a small spatial extent. This species may also be a flexible omnivore like its congener *G. falcata* [28], meaning that when it emerges from aestivation, it can feed on leaf litter that is immediately available in the stream following the summer leaf fall. The native fish species with which it shares these streams cannot survive in the absence of surface water and are predators, so they may suffer from starvation if they enter newly inundated streams, potentially further increasing fish mortality.

Although *G. oliros* is tolerant of harsh physicochemical conditions in stream pools [29], [30] it cannot withstand loss of surface water and so is likely to be lost from stream sections that dry frequently [22]. However, it is possible that *G. oliros* populations could persist in the perennially flowing sections of the regulated streams upstream of weirs in Camp, Gap and Brown Creek because it did so between 1979 and 1996, although population sizes declined during this time [13]. It may be unable to maintain small self–sustaining populations in headwater streams and hydrological connectivity may be required to allow recruitment to resupply populations [22], in particular because most *Galaxias* species are relatively short–lived. The size frequency data showed that most individuals were small, supporting the presence of younger fish in the unregulated streams.

*Galaxias maculatus* also tolerates high temperatures, low pH and low dissolved oxygen levels in stream pools [31], [32], and may even tolerate emersion for short periods [33]. It lives for ≈ 2 years [30], and is probably potamodromous in Rocklands Reservoir and its unregulated tributaries, migrating upstream to spawn in streams during spring flows, as has been observed for lake populations of this species in Western Australia [31], Victoria [32] and South America [34]: the presence of mostly larger fish in Cultivation Creek, where its abundances were highest, supports this notion. However, in the Wannon River it may be diadromous, but has never been recorded in the regulated tributaries upstream. Barriers against upstream dispersal are likely to be important in determining the mode of movement for reproduction in these native fish. While Camp and Number 1 Creeks flow into heavily vegetated wetlands, potentially isolating the headwaters from Rocklands Reservoir, Brown and Gap Creek flow into the Wannon River (which joins with the Glenelg many kilometres downstream of Rocklands). The historical and present day absence of this species might then be explained by obstructions in the lower and middle reaches, which are likely to prevent the migration of diadromous fish [35]. If this is the case, *G. maculatus* may not respond to further years of summer–autumn flow releases in these streams. Both galaxid fish clearly have a considerable capacity to survive drying in perennial stream pools, sufficient to withstand the millennium drought in the unregulated streams. Downstream of weirs, increased loss of stream pools due to extreme drying has probably caused local extinctions, while upstream of weirs, small populations in headwaters were probably not self–sustaining across decades in the absence of re-supply by migrating individuals.

*Gadopsis marmoratus* is a much longer–lived native fish and is territorial [36] and was sustained in the unregulated stream network by perennial pools. However, increasing temperatures as climate change progresses are likely to limit adult, juvenile and gamete survival of *G. marmoratus*, despite its tolerance of long periods without stream flow [37], [38]. Results suggest that it was recruiting in the unregulated streams. Abundances of this species in the unregulated streams were more consistent across times than the other species and its size frequency distributions were less skewed. *G. marmoratus* was also found both above and below the weirs in Brown Creek prior to 2006, but no individuals were collected during the present study. If *G. marmoratus* in Brown Creek were spring–summer breeders [36], [37], then, in the regulated streams prior to environmental flow releases, juveniles would have hatched when the stream channel was dry downstream of the weirs on both branches. As juveniles are probably the dispersing stage in this species [38] they may have been unable to leave their natal reaches during summer and may have been consumed by the territorial adults once they left the guarded nests, limiting both local and downstream recruitment. The known low fecundity in this species [30] may have assisted their decline prior to environmental flow releases. To recolonise the regulated streams, juveniles would need to be available downstream and able to move upstream into formerly dry reaches and through or over weirs, and this may be unlikely during spring–summer; although it is possible, as proven by the presence of a single juvenile collected downstream in Camp Creek in 2006.

### Persistence of native fish and crayfish in unregulated and regulated streams during supraseasonal drought

The fish assemblages in the unregulated streams have been remarkably consistent since they were first surveyed in 1979; although the fish fauna was not diverse, even for headwater streams, which typically have low alpha and higher beta diversity [39]. The consistent patterns observed during the present study differ from another long–term study of headwater stream fish, in Virginia, USA [7]. This study used similar methods to sample a more species-rich fish fauna in fewer streams, but over a longer time period (≈69 y), finding a high species turnover through time, especially in two streams which had been impacted by agriculture. The unregulated Victoria Range streams have not been impacted by development since 1979 and this may partially explain the temporal consistency of their assemblages. The presence of Rocklands Reservoir downstream might also act to prevent species turnover through limiting immigration. However, so few fish species occurred that it is difficult to assess turnover at decadal scales, with the exception of probable extinctions in the regulated streams. Nevertheless, the consistency of the differences in assemblages between these small streams, which was most marked at the height of the drought, is interesting, especially since the study reaches on different channels were often separated, through confluences, by only a few kilometres. At that time, there were conspicuous exclusions of species from different parts of the system: no galaxiids were found in Deep and Cultivation creeks; only a few *G. marmoratus* were collected from one pool in Cultivation Creek; a single *G. oliros* and few *G. marmoratus* were in Hut Creek; a single *G. oliros* was in Honeysuckle Creek (Table 1).

Regardless, the relative stability of the fish fauna in the unregulated streams suggests that native fish species coped fairly well with seasonal flows, and survived the millennium drought. Models have predicted increased abundances of *G. olidus* when cease–to–flow periods were reinstated in artificially perennial streams [19], and our results support the idea that the ecologically similar *G. oliros* tolerates seasonal drying in unregulated streams. In their review, Matthews and Marsh-Matthews [40] found that droughts often left few detectable effects on the fish fauna of the south-west USA. They suggested that climate change impacts may cause local extinctions through increased water temperature. Species distribution models for *G. olidus* showed varying results for different climate change scenarios [42], but some field observations suggest an increased frequency of local extinction in south–eastern Australia [41]. Species distribution models for *G. maculatus* showed minimal changes in distribution [42]. All three species also persisted in regulated streams with prolonged drying for several decades (regulation occurred between 1914 and 1960) prior to the onset of drought [13], [15]. It appears likely then, that the combination of the millennium drought and regulation led to the complete decline of native fish in the regulated streams.

### Relationship between fish and crayfish distributions

In the unregulated streams, high fish and crayfish counts did not overlap spatially. This pattern may be due to predation upon *Geocharax* by fish, especially by the larger *G. marmoratus,* since freshwater crayfish may form an important component of fish diets [43]–[45]. Therefore, in streams where fish abundances are lower due to prolonged drying, pools or dispersal barriers may comprise predation refuges for crayfish. Other studies have shown negative relationships between fish and crayfish and/or higher crayfish abundances in intermittent streams and wetlands, particularly following drought [46]–[49]. These patterns may have broader significance for other invertebrate species and detritus processing, because omnivorous crayfish have been shown to be ecosystem engineers in headwater streams, as well as direct predators on smaller invertebrates [50].

## Conclusions

A single year of summer–autumn environmental flow releases did not produce a clear, detectable response in fish or crayfish in headwater streams in the Victoria Range, Victoria, Australia. Strong evidence suggests that fish became extinct both upstream and downstream of weirs in four of these streams, between 1996 and 2008. Therefore, a detectable response to environmental flow releases would require recolonization of the streams, and this was not observed. Possible explanations for this include: 1) a single year of flow releases probably did not provide sufficient time for recolonisation by fish, in contrast to invertebrates which had not become extinct upstream of weirs; 2) the volume of flow releases did not provide sufficient flow for fish to migrate from downstream reaches; 3) dispersal barriers, including breaks in surface flow, may exist downstream of the weirs that prevent recolonization. For crayfish, the rate of dispersal is probably too slow to have allowed significant increases in density across all regulated streams during this study. An indefinite continuation of flow releases annually, and reducing the unnatural breaks in flow imposed by weirs, may eventually facilitate dispersal up regulated channels. But, other potential barriers also need to be identified and ameliorated, to provide the best conditions for fish and crayfish to fulfil their life cycles in these streams. Environmental flow releases in headwater streams should be accompanied by the identification and management of anthropogenic barriers to fish dispersal. Furthermore, the probable extinction of fish in the regulated streams but not in the unregulated streams, in response to decadal-scale drought, shows that human alterations to stream flow regimes may pose a greater threat than long-term drought or climate change impacts on flow regime.
